# Knowledge and barriers on correct use of modified guidelines for active management of third stage of labour: a cross sectional survey of nurse-midwives at three referral hospitals in Dar es Salaam, Tanzania

**DOI:** 10.4314/ahs.v20i4.49

**Published:** 2020-12

**Authors:** Fatina B Ramadhani, Yilan Liu, Melania Menrad Lembuka

**Affiliations:** 1 Nursing Department of Tongji Medical College, Huazhong University of Science and Technology; 2 Nursing department, Union Hospital of Tongji Medical College, Huazhong University of Science and Technology; 3 Clinical Nursing department, School of Nursing, Muhimbili University of Health and Allied Sciences (MUHAS); 4 Surgery department, Muhimbili National Hospital (MNH)

**Keywords:** Nurse-midwives' knowledge, maternal bleeding, maternal mortality, third stage of labour, AMTSL, atonic uterus

## Abstract

**Background:**

Despite the fact that it is possibly preventable, postpartum haemorrhage (PPH) is the global most deadly form of obstetric bleeding, mainly sub-Saharan Africa with at least one-fourth of maternal deaths in East African regions. Active management of third stage of labour (AMTSL) is recommended to prevent PPH. However, AMTSL guidelines have been revised since 2006.

**Objectives:**

To examine the current status of nurse-midwives' knowledge on modified AMTSL guidelines and highlight barriers to AMTSL correct use.

**Method:**

Descriptive cross sectional survey was conducted to 160 nurse-midwives at three referral hospitals in Dar es Salaam, Tanzania. One-way, interactive modes ANOVA and Chi square (χ2) test were run in SPSS 21 version to compare the association of independent and dependent variables.

**Results:**

Virtually all nurse-midwives knew the first recommended uterotonic (99.4%) and delayed cord clamping (98.8%) protocols as modified. Knowledge was significantly contributed by multiple factors; p=0.001. Reported correct AMTSL use was 46.8% which was significantly affected by AMTSL training (χ2 = 6.732, p = 0.009) and prioritizing atteding an asphyxiated baby (χ2 = 5.647, p = 0.017).

**Conclusion:**

Regardless of high nurse-midwives' AMTSL knowledge; it is imperative that responsible authorities plan appropriate strategies to solve reported barriers affecting correct AMTSL use.

## Introduction

Approximately 280,000 maternal deaths occur each year mainly in low and middle-income settings (LMICs)^1^,^2^. Despite the fact that it is largely preventable, postpartum haemorrhage (PPH) i.e (>500 mL of blood loss) and severe PPH (>1000 mL of blood loss) is the most deadly form of obstetric bleeding^3^. Nearly 2% globally and up to 10.5% of mothers in sub-Saharan Africa, experience PPH^4^ while at least one-fourth of maternal deaths among East African regions^3^,^5^,^6^. Third stage of labour (TSL) is the most critical period for PPH which is defined as the time from the birth of the baby until the expulsion of the placenta and membrane. PPH can be derived from uterine atone, lesions in the birth canal, uterine rupture, retained placental tissue or bleeding disorders^7^. However, uterus to contract and retract following child birth has been the most common cause of PPH. Active management of third stage of labour (AMTSL) is an evidence-based, low-cost intervention used to prevent PPH. Compared to the traditional approach “expectant” i.e. removal of the placenta by gravity, maternal effort and endogenous oxytocin stimulation not facilitated by uterotonic drugs; “AMTSL” significantly reduces PPH, decreases blood loss and the need for blood transfusions^8^. According to International Confederation of midwives (ICM) and International Federation of Gynaecology and Obstetrics (FIGO) 2003 definitions; the original AMTSL guidelines had three components. Those were (1) administration of uterotonic agents and cord clamping within one minute of child birth, (2) active removal of the placenta by controlled cord traction (CCT) following signs of placenta separation and (3) uterine massage (UM) immediately after removal of the placenta and consequently every fifteen minutes in first two hours of child birth^9^. In 2006 revised guidelines recommended delayed cord clamping (i.e. 1 to 3 minutes after birth) to allow a prolonged blood flow thus may improve iron status in the infant.

In 2012, WHO revised the definition to emphasize provision of uterotonic (preferably oxytocin)10 that should be done by a skilled child birth provider. But for uncomplicated child births CCT and sustained uterine massage are no longer recommended. Moreover, abdominal palpation was recommended in all women for early identification of postpartum uterine atone and continued monitoring of the uterus for 2 hours after birth, with fundal massage if the uterus is atonic. So far AMTSL is the main PPH prevention measure recommended by WHO for all child births, in LMIC settings^10^.

Despite of its effectiveness and benefits to prevent PPH, the use of AMTSL was previously reported low in Tanzania and other East Sub-Saharan countries^11^. However, with pre- and in-service trainings of key providers the knowledge and practice on recommended components were eventually improving though with some challenges including shortage of skilled birth attendants^12^. WHO had been doing several modifications of AMTSL guidelines and introduced new recommendations as discussed in other studies^13^. Tanzania is among countries that follow WHO guidelines in implementing AMTSL. This demanded for an assessment of the current status of the providers of child birth's knowledge and use of AMTSL in Tanzania.

In Tanzania normal childbirth services at the clinical settings are mainly conducted by skilled midwives who are also licenced nurses hence the word “nurse-midwives” has been used in this study. According to ICM ‘midwife’ is a person who has successfully completed a nursing-midwifery education programme that is based on the ICM essential competencies for basic nursing-midwifery practice and the framework of the ICM global standards for nursing-midwifery education and is recognized in the country where it is located; who has acquired the requisite qualifications to be registered and/or legally licensed to practice nursing-midwifery and use the title ‘midwife’; and who demonstrates competency in the practice of nursing-midwifery^14^. Tanzania Nursing and midwifery Act. 2010; defines “midwifery” as means of giving care and supervision to a woman during pregnancy, labour and postpartum period and caring for the new born babies and infants. To practice nursing-midwifery requires registration and license from Tanzania Nurses and Midwives Council (TNMC) as per regulations made under the Act^15^. The current nursing/midwifery training system of Tanzania produces enrolled nurse/midwives (have certificate) and registered nurse/midwives (have diploma or above) nursing/midwifery levels of education whom both qualify to work in midwifery settings. Prerequisites for these trainings need pass marks of ordinary level for secondary education as set by TNMC or an authority with the mandate for entry qualifications of nursing/midwifery programmes. Trainings can be conducted at school, college, institute or any other recognized place running nursing/midwifery education approved by the Council under the provisions of the Act, to provide a course leading to the acquisition of a qualifying award. Nurse-midwives being the majority of key health care providers in assisting normal child births at the clinical settings in Tanzania; we therefore examined their knowledge and highlight factors contributing to their knowledge and correct use of 2012 WHO modified AMTSL guidelines. Findings of this study are expected to bring light in improving child birth services, maternal health and reduction of maternal mortality in Tanzania.

## Methods

### Design and setting

Descriptive cross sectional study comparing within study participants was conducted from January to March 2018 at the maternity units of Amana, Mwananyamala and Temeke referral hospitals. These are major multi-service government funded referral hospitals in Dar es Salaam region. The region is the most populated city in Tanzania. Studied facilities provide 24 hours services by skilled nurse-midwives who are responsible for assisting most of vaginal child births while obstetricians' and other medical officers' roles focused on emergency and surgical obstetric care.

### Participants, inclusion and exclusion criteria

The study targeted nurse-midwives who had potential responsibility in helping women during child birth in three large urban referral hospitals. However, those with administrative responsibilities or were not working in either prenatal, intra-natal or postnatal units were excluded from the study.

### Sample

Convenient sampling was used due to the fact that consent to participate depended on the returning of the distributed questionnaires to eligible participants. The sample-size calculation based on other African studies where AMTSL knowledge and use was reported^18^,^19^. The minimum required sample size was estimated to be 160 nurse-midwives with 5% marginal error and a 95% confidence interval and 10% additional of none response rate.

### Instrument

A structured questionnaire was designed from a large body of literature especially from the revised ICM/FIGO and WHO AMTSL guidelines to address research objectives. Mainly the study measured the key components of AMTSL, which is defined by the 2012 WHO guidelines^10^. These include the use of uterotonic drug during the third stage of labour, appropriate dose and route of administration (if applicable), and indications for CCT or UM. Hence similar questions for current AMTSL components have been derived from several studies. These include type of first line uterotonics, timing, route and dose of injecting such uterotonic, timing for cord clamping, and miscellaneous updated advice like harmful practices to avoid during AMTSL^13^,^16^,^17^. Data collection tool was tested for reliability and validity with Cronbach's alpha coefficient 0.80. The questionnaire was comprised of; demographic data (age, sex, level of normal and nursing/midwifery profession education, category of local ministry nursing/midwifery cadre, and years of work experience of attending women in labour and child birth). The second part asked nurse-midwives about AMTSL training information like place of training whether they leaned it pre-service at formal nursing/midwifery training schools or they acquired it during in-service. They were also asked if aware of AMTSL and used it correctly. The third part included nine (9) specific multiple choice knowledge questions on current AMTSL practice with a total of nine scores. Each correct response carried one score while zero mark was for other options. Major questions included: purpose of AMTSL, first line recommended uterotonic; its dose, route, timing of uterotonic injection and cord clamping whether immediate or delayed duration, harmful practices on AMTSL events, indications for CCT and intervals for UM if necessary in postpartum. Participants were supposed to choose one correct answer or specify other answer(s). Finally participants had an opportunity to respond to an open ended question about factors that hinder their correct use of AMTSL when helping women in TSL. This paper mainly assessed the cognitive level of knowledge. However, the affective level (AMTSL correct use) couldn't be observed due to unavoidable reasons hence was measured theoretically through closed ended questions addressed in the questionnaire of this survey.

### Data collection

Data was collected from 160 nurse-midwives. The study was approved by Muhimbili University of Health and Allied Sciences research and ethics committee (reference No. MU/PGS/SAEC/Vol.XI/74). Also permit was obtained from the regional hospital authorities at all levels. Furthermore, informed verbal consent was requested from eligible participants. In addition returned completed questionnaire was considered indicative of participant's consent to participate. Ward in-charges assisted the distribution of questionnaires to eligible nurse-midwives who volunteered to participate in this survey. Data collected were anonymous and confidential. Participant had to complete the filling of questionnaire under supervision of researchers during break time within their normal working hours. This approach brought flexibility of data collection and minimized contamination of participant's pre-existing AMTSL knowledge as well. Additionally data were checked every day for completeness and accuracy.

### Data analysis

Data were recorded, cleaned, and analysed by using the Statistical Package for the Social Sciences (SPSS), version 17 (SPSS Inc., Chicago, IL, USA) then presented in frequency tables, bar and pie charts. Descriptive and inferential statistics were run for the association of variables. The first part of analysis used One-way and interactive modes ANOVA for the association of knowledge scores (continuous dependent) with demographics and AMTSL training information as (categorical independent) variables. The second analysis used Chi-square (χ2) test for the association of independent variables (demographics, AMTSL training information, reported barriers and grouped knowledge scores) with dependent (correct AMTSL use) categorical variables. A “p-value” less than 0.05 was regarded statistical significant result.

## Results

### Participants' knowledge on AMTSL

Response rate was 100%. Total knowledge correct scores for each of 9 items are presented in Table 1. Nurse-midwives demonstrated (100%) awareness and (94.4%) knew that AMTSL reduces postpartum haemorrhage. Virtually all (99.4%) knew oxytocin as first recommended uterotonic including its, dose (98.8), route (99.4), injection timing (96.3) and timing for cord clamping (98.8%). Majority (96.9%) knew that ignoring the assessment of uterus tonus, placenta completeness, cervical and vaginal tears for repair are harmful practices during AMTSL (Table 1).

**Table 1:** Nurse-midwives' total knowledge scores about AMTSL

Items (correct response)	n	%	Mean±SD
AMSL is recommended to (prevent PPH in third stage of labour (a))	151	94.4	0.94.±0.23
First line recommended uterotonic is (oxytocin (b))	159	99.4	0.99.±0.08
The recommended dose of first line uterotonic is (10IU (b))	158	98.8	0.99±0.11
Recommended route of first line uterotonic is (IV/IM-preferably intramuscular (IM) unless otherwise (c) )	159	99.4	0.99.±0.08
Cord clamping can be done within (1minute if immediate or 3 minutes if delayed (a))	158	98.8	0.99.±0.11
Uterotonic can be injected within (1minute if immediate or 3 minutes if relaxed (a))	154	96.3	0.96.±0.19
Believed harmful practice (s) on AMTSL (Ignore assessing uterine tonus/or placenta and cervical-vaginal tears (c))	155	96.9	0.97.±0.18
For uncomplicated deliveries CCT and sustained uterine massage (UM) are no longer recommended (True (a))	155	96.9	0.97.±0.18
In the first two hours post-delivery; interval of uterine massage if necessary is (15 minutes (a))	155	96.9	0.97.±0.18
**Mean knowledge score**	156	97.53	8.78.±0.82

**n-frequency** of correct responses per item, **SD-Standard Deviation**

**Figure 1:**
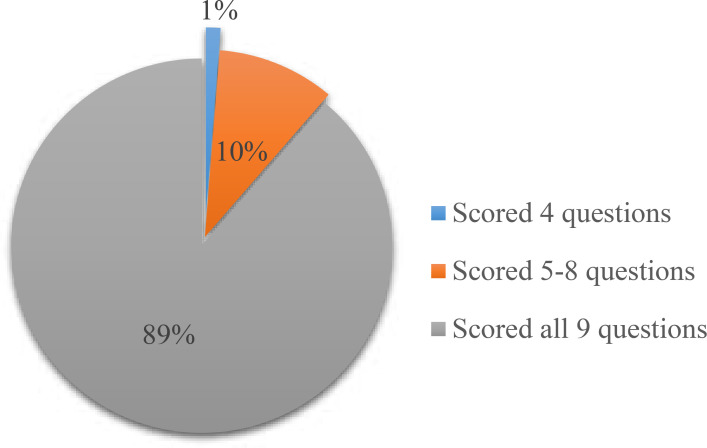
Total AMTSL questions scored correctly by nurse-midwives

Generally nurse-midwives demonstrated high knowledge for each question ranging from 94.4–99.4% with an average total score; M±SD= 8.78±0.82, while (89%) scored right all 9 items of knowledge questions with only 1% scoring less that 50% (Fig. 1).

### Factors related to nurse-midwives' knowledge of AMTSL

Table 2 presents demographic and AMTSL training factors of nurse-midwives and ANOVA statistical tests results for their association with total knowledge scores. Majority were females (141%), age ranged 22–59 years with diploma nursing-midwifery education (69%). Most received AMTSL training especially from nursing/midwifery training (94%). Results show that there was no enough evidence for the association of individual nurse-midwives' demographic or training variables with their knowledge of AMTSL (p>0.05) until all were interacted together (p=0.001). Therefore this association is multi-factorial.

**Table 2:** Differences in demographics and AMTSL total knowledge scores of nurse-midwives

Variables	Group (N=160)	Mean±SD	*P*
Age in years	21–30(47)	8.77±0.94	0.981
	31–40(63)	8.78±0.77	
	41–50(36)	8.75±0.87	
	51–60+(14)	8.86±0.36	
Sex	Male(19)	8.63±1.21	0.416
	Female(141)	8.79±0.75	
Highest educational level	College(125)	8.79±0.78	0.620
	University(35)	8.71±0.96	
Highest Nursing/midwifery qualification	Certificate(46)	8.78±0.79	0.624
	Diploma(69)	8.84±0.59	
	Advanced diploma(10)	8.50±1.58	
	Degree(35)	8.71±0.96	
Ministry Nursing/midwifery cadre	Enrolled midwife(46)	8.78±0.79	0.941
	Registered midwife(114)	8.77±0.83	
Clinical nursing-midwifery experience in years	1(63)	8.86±0.72	0.491
	2–5(52)	8.77±0.78	
	5+(45)	8.67±0.98	
Place of AMTSL training	Nursing/midwifery school(94)	8.78±0.82	0.977
	On job refresher course(66)	8.77±0.82	
Multiple effect of demographics	All demographics (160)	7.91±0.86	0.001*

* P<0.05

### Nurse-midwives' self-reported correct use of AMTSL and related factors

Fig. 2 illustrates nurse-midwives self-report on correct use of AMTSL. Nearly half (46.8%) indicated correct use of AMTSL during TSL. Table 3 shows differences in demographics and barriers on nurse-midwives' self-reported correct use of AMTSL. The association between independent categorical variables (demographics, AMTSL training information and barriers) with dependent categorical variable (AMTSL use) was tested by using χ2 test. A total of seven (7) factors were listed as major barriers when using AMTSL. Of these “Work overload”=insufficient staff coverage ratio and inadequate child birth space and equipment emerged as barriers to more than 90% of participants while being a novice midwife was a challenge of few 20(26.7). There was no much statistical differences between social demographics and barriers with correct AMTSL use except for the place where AMTSL was learned (χ2 = 6.732, p = 0.009) and prioritizing in saving the life of an asphyxiated new-born baby (χ2 = 5.647, p = 0.017) (Table 3).

**Table 3:** Differences in demographics and barriers on nurse-midwives' self-reported correct use of AMTSL

	N=160			

Variables	Correct (%)	No/don't know (%)	χ^2^	P
Age in years				
21–30	20(26.7)	27(31.8)	4.148	0.246*
31–40	30(40.0)	33(38.8)		
41–50	15(20.0)	21(24.7)		
51–60+	10(13.3)	4(4.7)		
Sex				
Male	11(14.7)	8(9.4)	1.051	0.305
Female	64(85.3)	77(9.6)		
Highest educational level				
College	61(81.3)	64(75.3)	0.850	0.356
University	14(18.7)	21(24.7)		
Highest Nursing/midwifery qualification				
Certificate	22(29.3)	24(28.2)	1.398	0.706
Diploma	33(44.0)	36(42.4)		
Advanced diploma	6(8.0)	4(4.7)		
Degree	14(18.7)	21(24.7)		
Ministry nursing/midwifery cadre				
Enrolled midwife	22(29.3)	24(28.2)	0.023	0.878
Registered midwife	53(70.7)	61(71.8)		
Clinical nursing-midwifery experience in years				
1	26(34.7)	37(43.5)	1.323	0.516
2–5	26(34.7)	26(30.6)		
5+	23(30.7)	22(25.9)		
Place of AMTSL training				
Nursing/midwifery school	36(48.0)	58(68.2)	6.732	0.009*
On job refresher course	39(52.0)	27(31.8)		
“Work overload”=insufficient staff coverage ratio				
Barrier	68(90.7)	77(90.6)	0.001	0.986
Not barrier	7(9.3)	8(9.4)		
Inconfidence due to varying AMTSL updates				
Barrier	65(86.7)	71(83.5)	0.308	0.579
Not barrier	10(13.3)	14(16.5)		
Being a novice nurse-midwife				
Barrier	20(26.7)	22(25.9)	0.013	0.910
Not barrier	55(73.3)	63(74.1)		
Inadequate delivery beds and kits				
Barrier	73(97.3)	81(95.3)	0.459	0.498
Not barrier	2(2.7)	4(4.7)		
Prioritizing an asphyxiated baby over AMTSL				
Barrier	54(72.0)	74(87.1)	5.647	0.017*
Not barrier	21(28.0)	11(12.9)		
Stressful work environment				
Barrier	63(84.0)	72(84.7)	0.015	0.902
Not barrier	12(16.0)	13(15.3)		
Fear of unknown child birth adverse outcome				
Barrier	61(81.3)	63(74.1)	1.190	0.275
Not barrier	14(18.7)	22(25.9)		
Knowledge on AMTSL				
Scored 4–8 questions	1(1.3)	4(4.7)	1.497	0.221
Scored all 9 questions	74(98.7)	81(95.3)		

* P<0.05

## Discussion

This study establishes evidence around providers specifically with nurse-midwives knowledge preparedness, and surrounding barriers on the correct use of revised AMTSL guidelines for PPH prevention. We found important high knowledge findings where by 100% of nurse-midwives were aware of AMTSL and knew that it is recommended for preventing PPH. The most key knowledge was also high on the most important component of AMTSL (99.4%) i.e. first recommended uterotonic (oxytocin) as well as other major components of AMTSL. This is an impressive finding and goes in line with thWHO current AMTSL guidelines and recommendations. This finding is similar to previous results where oxytocin uterotonic of choice was known by (99.8%), with a dosage 10 IU by (79.8%) and an intramuscular route by (100%) of midwives^20^. High rate of knowledge on uterotonic component was found in other settings^16^,^17^,^19^. Delayed cord clamping as currently recommended was also well known by most participants of this study. Furthermore, the current study found that palpation of the abdomen for early detection of an atonic uterus, assessment for placenta completeness and inspecting for cervical or vaginal tears were deemed important and become harmful if not observed when performing AMTSL. Other studies found similar observation on delayed cord clamping as a component of AMTSL but mixed findings on consideration of CCT and or UM^7^,^19^,^21^,^22^.

Factors associated with high knowledge in this survey were contributed by a combination of variables including demographics and the place of AMTSL training (whether acquired in-service or pre-service). This is similar to other study where by profession and year of graduation were the factors associated with obstetric care provider's knowledge towards AMTSL while pre/in service training was associated with obstetric care provider's practice^23^. Other findings in rural referral hospital in Northern Tanzania revealed that knowledge on help mothers survive bleeding after birth (HMS BAB) improved following training. However, that knowledge and simulated basic child birth skills decayed after nine months, while confidence and simulated obstetric emergency skills were largely rtained. Hence authors indicated a need for continuation of training and that future research should focus on the frequency and dosage of follow-up training^24^.

Regardless of very high knowledge on all components of revised AMTSL guidelines, self-reported correct use of AMTSL was just found to 47.88% of this study participants. This finding is disappointing due to un-matching seen with high knowledge found. However, this proportion is still higher than what was reported by nurse-midwives in Busegera district, Rwanda (15.9%)^25^ but less than large maternity hospital in Tirana, Albania where which was evaluated to few obstetricians 78% (21/27) who reported always or usually using AMTSL^26^. According to Stanton et al. (2009) the practice of AMTSL by obstetric care providers was also not satisfactory in Ethiopia. In contrast AMTSL practice was better in Ethiopia and Nigeria^11^.

Other researchers revealed that prophylactic administration of uterotonics directly after childbirth significantly increased in last decades from 1995 to 2011 by both midwives (10–59.1%) and obstetricians (55–96.4%) (p=0.01). It was further perceived as an essential part of AMTSL among midwives in Netherlands, but surprisingly was not standard practice in the low-risk population supervised by midwives^20^. As in Columbia correct use of AMTSL was found low at the largest maternity teaching centre according to FIGO/ICM (0.8%), WHO (0.0), and the Cochrane Collaboration (0.83%) description. These discrepancies called for an urgent need for a single definition of AMTSL, which could be used globally for research, training, and scaling-up the performance of AMTSL.

Individual components of AMTSL especially uterotonics (oxytocin) was well performed in public tertiary obstetric centres in southwest, Nigeria, but the rate of adherence to the international recommendations was low^18^. Low use of AMTSL as defined by international organizations was also reported at the largest obstetric teaching centre in Colombia^16^ and in three government-funded healthcare facilities in Uganda^17^ which are both consistent with response of nurse-midwives in the present study. These unresolved variations might reduce nurse-midwives' confidence and motivation in helping mothers in TSL.

On the other hand, regardless the reported fact that oxytocin was readily available in almost all child birth facilities in Tanzania^27^,^28^, the current study reveals that nurse-midwives faced several barriers in the correct use of AMTSL. Those factors include “Work overload”=-insufficient staff coverage ratio, in confidence due to varying AMTSL updates, being a novice midwife, inadequate child birth space and equipment, prioritizing saving the life of an asphyxiated new-born baby over AMTSL, stressful work environment and fear of unknown child birth adverse outcome. Some of these barriers are consistent with midwives' challenges for implementing AMTSL in other African nations as in Ghana^13^. In Uganda healthcare system issues; current knowledge, awareness, and use of clinical guidelines; and healthcare practitioner attitudes to updating their clinical practice were three major factors influencing the uptake of evidence-based practice^17^. Moreover, contextual factors like high value placed on pain during labour; and lack of consistent and correct knowledge regarding safe storage, dosing, and administration of oxytocin promoted inappropriate use of uterotonic in Bagalkot and Hassan districts, India^29^. In contrast interest, fear for being held personally responsible for outcomes triggered active intervention in second stage of labour, even if there was no indication to intervene^30^. However we found two significant barriers that could explain poor use of AMTSL. Challenges of correct AMTSL. These include training backgrounds of nurse-midwives and emergency circumstances that occur concurrently with AMTSL like a need to resuscitate an asphyxiated new-born during TSL (p < 0.05). Shortage of staff might be a contributing factor of abandoning AMTSL for other important child birth emergencies. Training variations could be due to lack of harmonized curricula for nursing-midwifery trainings in higher levels, varying AMTSL guidelines and low speed in searching clinical updates. Similar findings observed that staff undertaking multiple tasks may not have enough time to follow the protocol^27^ and health facility factors significantly influence utilization of AMTSL^31^. On the other hand provider preparedness and facility readiness to deal with institutional births and associated complications had successful improved just through onsite nurse mentoring programmes^32^. Quality in health care has been recognized as essential for well-being of the population and as a basic aspect of human rights^19^. Therefore it is imperative to empower health providers with necessary knowledge, appropriate work facilities and supportive supervision in order to achieve health care of high quality.

The strength of this study is that, we were able to compose questions that would capture nurse-midwives' knowledge on common understanding and most important series of AMTSL components as per WHO guidelines especially on uterotonic and cord clamping. We also got 100% response rate by participants. So we believe the structured questionnaire used in our study might guide other instruments for other settings related assessment. However, as part of our future plan we expect to conduct an observational study that would assess real AMTSL events to get the actual current picture of AMTSL performance in the clinical settings.

We were limited by the use of convenient sample so if midwife response on whether used AMTSL correctly or not; answers given by the respondents were personal, not necessarily representative of the whole maternity department across the three hospitals. However, their answers were accompanied by reasons or barriers to poor performance hence if taken into consideration might contribute to positive changes in the respective and other applicable settings. Furthermore the expansion of sample size, study coverage and application of none-convenient sampling methods might improve generalizability of findings in future related study.

## Conclusion

We found that, nurse-midwives are aware and demonstrated high knowledge on the current AMTSL guidelines. Their knowledge was associated with a combination of factors including demographics and the place of AMTSL training (whether acquired in-service or pre-service). Furthermore nurse-midwives face barriers regarding correct use of AMTSL. The most significant factor was interference of child birth emergencies like rushing to save the life of an asphyxiated born child. This study observed that having trainers who are using varying AMTSL guidelines might hinder trainee's confidence and performance. Hence there is necessity of steady and speedy sharing of evidenced updates for common understanding regarding third stage management and nursing-midwifery practices in general. Also lives of the mother receiving AMTSL and an asphyxiated child are equally important that both need secial attention hence facilities and responsible authorities should improve child birth staffing to overcoming these challenges. It is also imperative to identify and find sustainable solutions of challenges faced in improving child birth services for the reduction of maternal mortality in Tanzania.

## References

[1] WHO (2014). Trends in Mternal Mortality: 1990–2013. Estimates by WHO, UNICEF, UNFPA, The World Bank and the United Nations Population Division.

[2] Alkema L, New JR, Pedersen J, You D, Bastian P, Wu J (2014). Child mortality estimation 2013: An overview of updates in estimation methods by the United Nations Inter-Agency Group for Child Mortality Estimation. PLoS One.

[3] Afnan-Holmes H, Magoma M, John T, Levira F, Msemo G, Armstrong CE (2015). Tanzania's Countdown to 2015: An analysis of two decades of progress and gaps for reproductive, maternal, newborn, and child health, to inform priorities for post-2015. Lancet Glob Heal.

[4] Ononge S, Mirembe F, Wandabwa J, Campbell OMR (2016). Incidence and risk factors for postpartum hemorrhage in Uganda. Reprod Health.

[5] Melkert P, Melkert D, Kahema L, Van Der Velden K, Van Roosmalen J (2015). Estimation of changes in maternal mortality in a rural district of northern Tanzania during the last 50 years. Acta Obstet Gynecol Scand.

[6] Pembe AB, Paulo C, D'mello BS, van Roosmalen J (2014). Maternal mortality at muhimbili national hospital in Dar-es-Salaam, Tanzania in the year 2011. BMC Pregnancy Childbirth.

[7] De Castro Parreira MVB, Gomes NCF (2013). Preventing postpartum haemorrhage: Active management of the third stage of labour. J Clin Nurs.

[8] Am C, Ness A, Je T (2010). Prophylactic oxytocin for the third stage of labour (Review). Cochrane Libr.

[9] ICM/FIGO (2003). Management of the third stage of labour to prevent postpartum hemorrhage: international joint policy statement. Jogc.

[10] World Health Organization (2012). WHO recommendations for the prevention and treatment of postpartum haemorrhage.

[11] Stanton C, Armbruster D, Knight R, Ariawan I, Gbangbade S, Getachew A (2009). Use of active management of the third stage of labour in seven developing countries. Bull World Health Organ.

[12] Raams TM, Browne JL, Festen-Schrier VJMM, Klipstein-Grobusch K, Rijken MJ (2018). Task shifting in active management of the third stage of labor: A systematic review. BMC Pregnancy Childbirth.

[13] Schack SM, Elyas A, Brew G, Pettersson KO (2014). Experiencing challenges when implementing Active Management of Third Stage of Labor (AMTSL): A qualitative study with nurse-midwives in Accra, Ghana. BMC Pregnancy Childbirth.

[14] International Confederation of Nurse-midwives Definition of the Midwive.

[15] Tanzania Nurses and Nursing-midwifery Council (2010). The Nursing and Nursing-midwifery Act.

[16] Miranda JE, Rojas-Suarez J, Paternina A, Mendoza R, Bello C, Tolosa JE (2013). The effect of guideline variations on the implementation of active management of the third stage of labor. Int J Gynecol Obstet.

[17] Braddick L, Tuckey V, Abbas Z, Lissauer D, Ismail K, Manaseki-Holland S (2016). A mixed-methods study of barriers and facilitators to the implementation of postpartum hemorrhage guidelines in Uganda. Int J Gynecol Obstet.

[18] Oladapo OT, Fawole AO, Loto OM, Adegbola O, Akinola OI, Alao MO (2009). Active management of third stage of labour: A survey of providers' knowledge in southwest Nigeria. Arch Gynecol Obstet.

[19] Lazzerini M, Ciuch M, Rusconi S, Covi B (2018). Facilitators and barriers to the effective implementation of the individual maternal near-miss case reviews in low/middle-income countries: A systematic review of qualitative studies. BMJ Open.

[20] Smit M, van Stralen G, Wolterbeek R, van Dillen J, van Roosmalen J, Slootweg Y (2013). Survey of prophylactic use of uterotonics in the third stage of labour in the Netherlands. Nursing-Midwifery [Internet].

[21] Boere I, Smit M, Roest AAW, Lopriore E, Van Lith JMM, Te Pas AB (2015). Current practice of cord clamping in the Netherlands: A questionnaire study. Neonatology.

[22] Prick BW, Vos AA, Hop WCJ, Bremer HA, Steegers EAP, Duvekot JJ (2013). The current state of active third stage management to prevent postpartum hemorrhage: A cross-sectional study. Acta Obstet Gynecol Scand.

[23] Tenaw Z, Yohannes Z, Amano A (2017). Obstetric care providers' knowledge, practice and associated factors towards active management of third stage of labor in Sidama Zone, South Ethiopia. BMC Pregnancy Childbirth.

[24] Nelissen E, Ersdal H, Østergaard D, Mduma E, Broerse J, Evjen-Olsen B (2014). Helping mothers survive bleeding after birth: An evaluation of simulation-based training in a low-resource setting. Acta Obstet Gynecol Scand.

[25] Puri R, Rulisa S, Joharifard S, Wilkinson J, Kyamanywa P, Thielman N (2012). Knowledge, attitudes, and practices in safe motherhood care among obstetric providers in Bugesera, Rwanda. Int J Gynecol Obstet.

[26] Bimbashi A, Ndoni E, Dokle A, Duley L (2010). Care during the third stage of labour: Obstetricians views and practice in an Albanian maternity hospital. BMC Pregnancy Childbirth.

[27] Mfinanga GS, Kimaro GD, Ngadaya E, Massawe S, Mtandu R, Shayo EH (2009). Health facility-based active management of the third stage of labor: Findings from a national survey in Tanzania. Heal Res Policy Syst.

[28] Bishanga DR, Charles J, Tibaijuka G, Mutayoba R, Drake M, Kim YM (2018). Improvement in the active management of the third stage of labor for the prevention of postpartum hemorrhage in Tanzania: A cross-sectional study. BMC Pregnancy Childbirth.

[29] Deepak NN, Mirzabagi E, Koski A, Tripathi V (2013). Knowledge, Attitudes, and Practices Related to Uterotonic Drugs during Childbirth in Karnataka, India: A Qualitative Research Study. PLoS One.

[30] Miltenburg AS, Kiritta RF, Meguid T, Sundby J (2018). Quality of care during childbirth in Tanzania: Identification of areas that need improvement. Reprod Health.

[31] Felarmine M, Joachim O, Agina O (2016). Facility factors influencing utilization of active management of third stage of labour among skilled birth attendants in Kiambu county, Kenya. Pan Afr Med J.

[32] Jayanna K, Bradley J, Mony P, Cunningham T, Washington M, Bhat S (2016). Effectiveness of onsite nurse mentoring in improving quality of institutional births in the primary health centres of high priority Districts of Karnataka, South India: A cluster randomized trial. PLoS One.

